# Editorial: Novel strategies for controlling mosquito-borne diseases

**DOI:** 10.3389/fpubh.2023.1171634

**Published:** 2023-03-13

**Authors:** Sheng-Qun Deng, Yu Cai, Duo-Quan Wang

**Affiliations:** ^1^Department of Pathogen Biology, The Key Laboratory of Microbiology and Parasitology of Anhui Province, The Key Laboratory of Zoonoses of High Institutions in Anhui, School of Basic Medical Sciences, Anhui Medical University, Hefei, China; ^2^Laboratory Animal Research Center, School of Basic Medical Sciences, Anhui Medical University, Hefei, China; ^3^Temasek Life Sciences Laboratory, National University of Singapore, Singapore, Singapore; ^4^Department of Biological Sciences, National University of Singapore, Singapore, Singapore; ^5^National Institute of Parasitic Diseases, Chinese Center for Disease Control and Prevention (Chinese Center for Tropical Diseases Research), Shanghai, China; ^6^National Health Commission Key Laboratory of Parasite and Vector Biology, Shanghai, China; ^7^WHO Collaborating Center for Tropical Diseases, National Center for International Research on Tropical Diseases, Shanghai, China

**Keywords:** mosquito-borne diseases, malaria, Zika virus, COVID-19, mosquito control, dengue virus

Mosquitoes are responsible for various protozoal and viral diseases, such as malaria, filariasis, dengue, and Zika (1). Due to globalization, urbanization, and climate change, these diseases have spread globally and remain our most challenging high-mortality diseases (2). Finding a practical approach to attack these mosquitoes is necessary and significant to eradicate the threat of mosquito-borne diseases. However, since World War II, we have relied too heavily on chemical pesticides to control mosquitoes. The excessive reliance on chemical pesticides has led to the development of insecticide resistance, and pesticide residues pose a threat to human and non-target organisms (3, 4).

In addition, the COVID-19 pandemic presents new challenges to the control of mosquito-borne diseases. According to WHO's World Malaria Report 2021, there were an estimated 241 million malaria cases and 627,000 malaria deaths around the world in 2020 (5). Compared with 2019, the number of cases increased by approximately 14 million, and the number of deaths increased by 69,000 in 2020. About two-thirds of these new deaths are related to the interruption of malaria prevention, diagnosis, and treatment during the COVID-19 pandemic. Therefore, new agents and strategies to address these challenges are urgently needed.

Biological control, represented by the release of male mosquitoes [incompatible insect technique and sterile insect technique (6, 7)] and biopesticides [*Bacillus thuringiensis* (8), *Beauveria bassiana* (9–11), and *Metarhizium anisopliae* (12)], can solve many of the pain points of traditional vector measures in the current context. Moreover, several mosquito vector control technologies are still being developed, including acoustic larvicides (13), RNAi-based bioinsecticide (14), unmanned aerial vehicle spraying (15), and nanotechnology (16), which are also expected.

This Research Topic, “*Novel Strategies for Controlling Mosquito-borne Diseases*”, aimed to provide new insights into the control of mosquito-borne diseases. *Aedes* mosquitoes are considered to be the main vector of arboviruses in the urban environment. Among them, special attention should be given to *Aedes aegypti*, the primary vector of dengue, chikungunya, and Zika in several countries worldwide. In July and September 2017, Leandro et al. conducted a pilot study to improve the existing integrated surveillance system through entomo-virological surveillance to determine regional priorities and actively search for individuals with symptoms of arbovirus infection. They used local molecular biological facilities to screen arboviruses in captured *Aedes aegypti* mosquitoes and then carried out serological investigation on suspected cases of dengue/Chikungunya near the collection site of dengue virus/Chikungunya virus-positive mosquitoes. This study proved that detecting and serotyping arboviruses in mosquitoes and individuals with symptoms during active surveys could provide warning signals of early transmission of arboviruses.

In addition, *Aedes albopictus* is the primary vector of infectious diseases (dengue) transmitted by *Aedes* mosquitoes in China, which has caused public health concerns. A study by Wei et al. investigated the genomic patterns of the spatial population genetic structure of *Ae. albopictus* across China. The genome-wide single nucleotide polymorphisms (SNPs) revealed seven gene pools and a fine spatial genetic structure of the *Ae. albopictus* population in China. The fine spatial genetic structure and gene flow data based on genome-wide SNPs and other relevant factors, such as mosquito density, rainfall, and climate, will be valuable for mosquito monitoring and epidemiological prediction and modeling of the incidence rate and spread of mosquito-borne diseases.

Another study by Tahir et al. evaluated the impact of climate conditions on the distribution of mosquito species in Qatar. They used the Naive Bayes model to calculate the posterior probability for various mosquito species collected from multiple sites in Qatar. The result of the Naive Bayesian prediction was used to determine the favorable environmental conditions of mosquito species. They found that *Culex* spp. is the most abundant, followed by *Anopheles* and *Aedes* spp. in Qatar. Higher temperatures and lower humidity would increase the chances of these two species (*Anopheles* and *Aedes*). However, with the decrease in humidity, the risk of *Aedes* mosquitoes will increase but only be limited to a certain point and then decline. A relative humidity of 35 to 45% and temperatures of 35 to 40°C are ideal for *Aedes* mosquitoes. Their results reiterate the need for a robust monitoring system combined with environmental departments and a wide range of multivariate data sets to more clearly predict mosquito species' potential distribution and abundance.

In addition, *Plasmodium falciparum* (*Pf*) 5-aminolevulinic acid synthase (5-ALAS) is a critical enzyme with high selectivity during liver stage development, indicating its potential as a target of prophylactic antimalarial drugs. Oduselu et al. used pharmacophore modeling, virtual screening, qualitative structural assessment, *in silico* absorption, distribution, metabolism, excretion, and toxicity (ADMET) evaluation, and molecular dynamics (MD) simulation to identify crucial potential lead compounds that can be used as Pf 5-ALAS inhibitors. It was observed that compound CSMS00081585868 was the best target, with a binding affinity of −9.9 kcal/mol and a predicted Ki of 52.10 nM, and formed seven hydrogen bonds with the amino acid residues of the target active site. The *in silico* ADMET prediction revealed that all ten best hits had relatively good pharmacokinetic properties. The qualitative structural assessment of the best target CSMS00081585868 showed that the existence of two pyridine scaffolds significantly promoted their ability to have a strong binding affinity with receptors. The best hit also showed the stability of the active site of Pf 5-ALAS, as confirmed by the RMSD obtained during MD simulation.

In conclusion, the collection of articles highlights some crucial advances in controlling mosquito-borne diseases, which will contribute to developing and applying new vector control measures.

## Author contributions

All authors listed have made a substantial, direct, and intellectual contribution to the work and approved it for publication.
